# Home, but Left Alone: Time at Home and Child Abuse and Neglect During COVID-19

**DOI:** 10.1177/0192513X211048474

**Published:** 2023-02

**Authors:** Lindsey Rose Bullinger, Angela Boy, Megan Feely, Stephen Messner, Kerri Raissian, William Schneider, Shannon Self-Brown

**Affiliations:** 1 1372 Georgia Tech, Atlanta, GA, USA; 2 1367 Children’s Healthcare of Atlanta, Atlanta, GA, USA; 3 7712 University of Connecticut, Hartford, CT, USA; 4 Department of Public Policy, 7712 University of Connecticut, Hartford, CT, USA; 5 University of Illinois, Urbana-Champaign, Urbana, IL, USA; 6 1373 Georgia State University, Atlanta, GA, USA

**Keywords:** J12, J13, I18, child abuse, child neglect, COVID-19, public policy

## Abstract

We use high-frequency mobile phone movement data and quick-release administrative data from Georgia to examine how time at home during the COVID-19 pandemic is related to child maltreatment referrals. Findings show that referrals plummeted by 58% relative to previous years, driven by fewer referrals from education personnel. After this initial decline, however, each 15 minutes at home was associated with an increase in referrals of material neglect by 3.5% and supervisory neglect by 1%. Our results describe how children have fared during the initial wave of the pandemic, and the results have long-term implications for child development and well-being.

## Introduction

In the spring of 2020, state and local governments instructed nearly all Americans to stay home to slow the spread of Sars-Cov-2 (or the novel coronavirus known as COVID-19). These public health emergency declarations and stay-at-home orders led to a sudden and unprecedented increase in the amount of time people spent at home (Dave, Friedson, Matsuzawa, & Sabia, 2021; Gupta et al., 2020). The sudden closure of schools and daycares presented many parents with the challenge of simultaneously providing childcare and children’s educational instruction. Early on, many professional organizations expressed concern over how this change would affect parental employment, parent and child mental health, child access to school, and child maltreatment^1^ (Abramson, 2020; American Academy of Pediatrics, 2020).

In this paper, we describe and quantify the relationship between the abrupt and unprecedented increase in time at home, extensive job loss and enormous shifts in home-based responsibilities, and drastic reductions in child maltreatment referrals. Building on research on community context and maltreatment, we also investigate if the relationship was more pronounced in particular communities. We first find that, following the public health emergency declaration in Georgia, child maltreatment referrals fell by 58% relative to previous years, largely driven by education personnel. Once the new lower baseline was established, however, more time at home was associated with more referrals of child maltreatment. This relationship is driven by referrals of supervisory neglect (i.e., children left unattended) and material neglect (i.e., inability to provide material necessities). We find no relationship between time at home and other types of neglect or abuse. In light of the first finding that child maltreatment abruptly decreased our results likely represent a lower bound.

This study features two major advantages over current literature. First, we obtained detailed, weekly child maltreatment referral data. These data allow us to document changes by specific maltreatment types and reporter *and* enable us to more precisely measure changes in March 2020 through the initial reopening of the state. Most literature on the topic is limited to monthly data and/or very few distinctions between types of maltreatment, if any is made (Baron et al., 2020; Bullinger et al., 2020b, 2021). Second, by coupling these referral data with cell phone tracking data, we can dig beyond general trends in maltreatment referrals and uncover relationships between behavior changes as a result of the pandemic and child maltreatment risk. Together, these features offer a more nuanced picture of how children and families fared in the early stages of the pandemic.

These results offer important policy, program delivery, and research implications. First, they suggest that the lack of school and childcare options left children unsupervised and without access to basic necessities in the early stages of lockdown. The social policy response to such changes must be robust to assist families throughout the duration of this pandemic, the return to the “new normal”, and in subsequent waves or other public health crises. Second, these findings also imply that if child physical abuse increased as much as many child advocates warned (American Academy of Pediatrics, 2020), then it is going largely undetected by child protective services (CPS). Researchers need to account for the large drop in reporting, and offer creativity in measuring child maltreatment during and post-pandemic. For example, in addition to using cell phone tracking data as we have done, some researchers have used social media data in an attempt to overcome detection hurdles (Babvey et al., 2020).

Finally, beyond COVID-19 specific policies, child maltreatment has both short- and long-term consequences, leading to lower educational attainment, employment, mental and physical health, and increased criminal justice system involvement (Currie and Spatz Widom 2010; Currie and Tekin 2012; Fletcher 2009; Thornberry et al. 2010; Zielinski 2009). That we find increases in referrals with more time at home implies there will likely be detrimental effects for children that may extend far beyond the pandemic, and policymakers should be prepared to address these unique challenges. Incorporating the costs of failing to provide children with safe and consistent care (Peterson et al., 2018) will be important in considering the costs and benefits of responses to the current (and potentially future) pandemic.

## Child Maltreatment and the COVID-19 Pandemic

The term child maltreatment applies to a wide range of child abuse and neglect. While there are several different valid approaches to assessing child maltreatment, each has advantages and disadvantages (Font & Maguire-Jack, 2020) There are approximately six million official reports of child maltreatment annually (US Department of Health and Human Services, 2020 Child maltreatment reporters are often members of the community who witness a situation that they believe could be child maltreatment, and often reports are made by mandated professionals.

A report is made to a hotline where the situation is screened by a trained worker to determine whether additional investigation is needed. Screened-in reports are investigated or assessed and some of these reports will eventually be substantiated, that is a determination will be officially made that the event was likely or was not likely harmful or the child. Families may be offered services at any point along this process to try to prevent a future incident of maltreatment and to ameliorate possible adverse effects from the maltreatment. However, while a number of studies identify psychosocial or behavioral correlates of maltreatment, such as parental substance misuse, and many theories propose associations between these factors and maltreatment, a growing body of work supports a causal relationship between financial hardship (which is correlated with unemployment) and child maltreatment—specifically child neglect (Berger, Font, Slack, &Waldfogel, 2017; Cancian et al., 2013; Raissian & Bullinger, 2017). However, the impact of financial hardship on various subtypes of neglect is understudied (Bullinger et al., 2020a). For example, paid employment may lead to less material neglect (inadequate provision of basic necessities), but a greater risk of supervisory neglect (inadequate supervision). Importantly, neglect makes up about 70% of all referrals, with supervisory neglect comprising about 70% of all neglect referrals.

In March 2020, physicians raised concerns about pandemic-related parental stress causing severe physical abuse (Agrawal, 2020), leading the American Academy of Pediatrics to issue guidelines about the role of pediatricians in keeping children safe during the pandemic (American Academy of Pediatrics, 2020). Feely et al. (2020) warned that supervisory neglect might rise if parents had to choose between working and adequately supervising their children. Spikes in unemployment also created concerns about families’ ability to meet children’s material and physical needs (DeParle, 2020).

Importantly, the existing literature provides guidance on how child maltreatment might respond due to the economic shock of COVID-19, but because COVID-19 bombarded families with multiple stressors all at once. The relationship between COVID-19-induced time at home and child maltreatment may or may not conform to findings from prior work. Below, we discuss work germane to the unique situation caused by the COVID-19 pandemic and its policy response.

### Employment and Economic Hardship Among Parents as a Pathway to Maltreatment

During the COVID-19 pandemic, the United States experienced its highest unemployment rate in over 50 years (U.S. Bureau of Labor Statistics, 2020)^2^ This resulted in a drop in many household incomes. COVID-19 has also had a unique effect on employment: many parents retained their employment but were suddenly expected to work from home while simultaneously providing childcare. Parents may have provided this care themselves, relied on older siblings, or due to absence of care options, some children may have been left home alone. All of these scenarios could have resulted in a situation of supervisory neglect while a distracted or inexperienced adult was caring for children.

### Limited Options: School Closures, Educational Requirements, and Disappearing Childcare

The public health response to the COVID-19 pandemic created a childcare market failure; despite high demand, it was unavailable for purchase even for well-resourced parents (Ali et al., 2021) and home-provided care was the only option. Further, the contagious nature of the virus and populations with the highest risk of mortality (e.g., elderly people) made it difficult to rely on informal childcare providers, including grandparents and family members, to help with childcare. In the case of school-aged children, parents also needed to supervise at-home learning, many did so while they were working, which created additional time and attention pressures for parents (Chen et al. 2021; Toness, 2020).

Other studies document an increase in parental stress and in material need. Within 1 week of the federal social distancing guidelines being put into place, 15% of sampled American parents reported that they had increased discipline of their child since the pandemic began (Lee & Ward, 2020). Since the pandemic began, parents have reported deteriorating mental health, lower patience with their children, and heightened feelings of being overwhelmed by parenthood (Gassman-Pines, Ananat, & Fitz-Henley, 2020; Kalil, Mayer, & Shah, 2020). Moreover, many families could not access the nutritional and health services that many public schools provide (Bitler et al., 2020; Chen et al., 2021).

Finally, school and daycare closures mean children lost regular interaction with a common source for reporting child maltreatment: education personnel who are all mandated reporters. Previous research suggests that school closures are a primary reason for fewer referrals during the pandemic (Baron et al., 2020); we also provide direct evidence of this phenomenon.

### The Current Study

In this study, we first examine whether COVID-19-related policies, including emergency declaration and school closures and affected child maltreatment referrals. We hypothesize that child maltreatment referrals decreased after the emergency declaration due to reduced interaction with mandated reporters. We propose two additional hypotheses designed to parse this trend.

1. After the public health emergency declaration, more time at home would be associated with increased material and supervisory neglect, relative to baseline rates. Although we are unable to test potential pathways, we theorize that (a) supervisory neglect increased as a result of employment changes and (b) material neglect increased as a result of income losses.
2. More time at home would be associated with increased physical abuse. Recently released empirical studies indicate that the pandemic may have increased important factors associated with physical abuse: increased mental health problems, substance use, and domestic violence (e.g., Bullinger et al., 2021; Leslie & Wilson, 2020; Sanga & McCrary, 2020) We are unable to test these pathways in our analyses, but they offer important context for our findings.

## Data

### Child Maltreatment Referrals

Despite the growing need to understand how COVID-19 and the early policy response affected a wide range of outcomes, relevant data are scarce and slow to emerge. In particular, real-time, publicly available, and nationwide child maltreatment data are not currently available.^3^ Instead, we use county-level referral data at the weekly level for the state of Georgia, obtained from the Georgia Division of Family and Children Services (DFCS). These data include allegations of child abuse or neglect, including material neglect, supervisory neglect, educational neglect, emotional neglect, medical neglect, physical abuse, and sexual abuse, for all 159 counties from January 2018 through May 4, 2020. These data reflect referrals prior to any decisions regarding screening, track assignment, or investigation. To compare 2020 over the same period in earlier years, we limit the analytic sample to the first 18 weeks of each year (*N* = 159 counties*18 weeks*3 years = 8586). Appendix A provides descriptions for each maltreatment type. To account for a county’s child population, we create a referral rate for each of the maltreatment types listed above for each county in each week, all rates are per 10,000 children.

### Cell Phone Tracking Data

We merge these county-week referral data with aggregate and anonymous cell phone tracking data from SafeGraph, Inc SafeGraph tracks 35 million unique mobile smartphone devices each month with exact known location in the United States. All 159 of Georgia’s counties are represented in the cell phone data. Each device is assigned a home location using its evening (6p.m. to 7a.m.) location. This location is defined by a 153-m by 153-m area that receives the most Global Positioning System (GPS) pings. The database then tracks the location of each device, indicating where it frequents, how long it stays, and the distance it travels, etc. in each day. Our primary measure is the percentage of the day that a device stays at its home location and a secondary measure is the percent of devices that are at home the entire day, i.e. where the person does not leave their house. The data are aggregated to the Census block group level.^4^ We further aggregate to the county-week level to examine the relationship between social distancing (as measured by time spent at home) and child maltreatment referrals.^5^ These data have been used in recent analyses examining the effect of state government restrictions due to COVID-19 (e.g., Dave et al., 2021; Friedson, McNichols, Sabia, &Dave, 2020; Gupta et al., 2020).

## Methods

The first goal of this study is to determine how child maltreatment referrals were affected by Georgia’s public health emergency declaration, and the state’s subsequent efforts encouraging people to stay home (e.g., closing schools). Georgia’s Governor declared a public health emergency on March 14, 2020 and ordered schools to close March 18, 2020, during the 10th week of the year. We compare trends in child maltreatment referral rates before and after the 10th week of 2020 relative to those over the same time period in 2018 and 2019, thereby estimating the effects of these policies. Specifically, we estimate the following equation

(1) $$Y_{cwy}=\alpha +\beta_{1}EmDec_{wy}+\delta_{c}+\tau_{w}+\;\gamma_{y}+\varepsilon_{cwy}$$

In equation (1), *Y* is the child maltreatment referral rate for county *c* in week *w* during year *y*. *EmDec* represents the effects of the COVID-19 emergency declaration equaling 1 if *w* is greater than or equal to 11 and *y* equals 2020, and zero otherwise. The coefficient of interest is $\beta_{1}$. We also include county fixed effects, $\delta_{c}$, which account for time-invariant characteristics of a county that may be correlated with child maltreatment referral rates, such as a county’s overall attitude regarding parenting strategies. This feature also allows us to compare trends within counties. Week fixed effects, $\tau_{w}$, account for overall trends in referrals that are similar across all counties, such as potential drops in referrals that occurred statewide. Finally, year fixed effects, $\gamma_{y}$, account for annual statewide changes affecting all counties, such as changes in DFCS policy, overall economic and political climate, etc. Due to the recency and disaggregated nature of the data, we are limited in our ability to include other potential confounders in the model. We study changes over time within counties, and account for statewide changes and seasonal patterns in referrals, which assuage this concern. All regressions are weighted by child population, and SEs are clustered at the county-week level to account for serial correlation.

We also present weekly event study figures to test the robustness of the research design and to consider how effects evolve over time. This event study model takes the following form

(2) $$Y_{cwy}=\sum_{\begin{array}{c} w=-9\\ w\neq 0 \end{array}}^{8}\beta EmDec_{cwy}+\delta_{c}+\tau_{w}+\;\gamma_{y}+\varepsilon_{cwy}\;$$

where the variables are the same as above, and β represents the coefficients of interest. This event study first allows us compare whether trends in referrals rates in the weeks leading up to the emergency declaration are statistically similar across years. These coefficients also allow us to track dynamic effects as the pandemic and the government’s response to it evolves.

The second goal of this study is to examine how time at home during the pandemic is related to child maltreatment referrals. To answer this question, we estimate the following model

(3) $$Y_{cw}=\alpha +\beta_{1}HomeTime_{cw}*Post_{w}+\beta_{2}HomeTime_{cw}+\beta_{3}Post_{w}+\delta_{c}+\tau_{w}+\varepsilon_{cw}\;$$

Here, we limit the analysis to weeks 1 through 18 of 2020 (*n* = 159 counties*18 weeks = 2862). This time period also includes the first 2 weeks of the state’s reopening (which occurred on April 24, during week 16). Importantly, although the state re-opened, schools remained closed. *HomeTime* represents the percent of time people within county *c* spent at home, on average, during week, *w*, as measured by smartphone movement. *Post* equals one for weeks 11 through 18 and zero otherwise. $\beta_{1}$ then shows the differential effect that time at home had after the emergency declaration, relative to before it in 2020. We also include county and week fixed effects, allowing us to both compare within counties and account for how the pandemic transpired throughout these weeks in Georgia. Regressions are weighted by child population, and SEs are clustered at the county-level. In addition to estimating equation (3) for the full sample of counties, we further determine if time at home during the pandemic had different relationships with child maltreatment referrals across county characteristics, including metropolitan status, percent of black residents, and poverty rate.

Finally, equation (4) estimates an event study for equation (3), where the percent of time spent at home interacts with a binary variable for each week, rather than a summary post variable.

(4) $$Y_{cw}=\sum_{\begin{array}{c} w=-9\\ w\neq 0 \end{array}}^{8}\beta HomeTime_{cw}*Week_{w}+\delta_{c}+\tau_{w}+\varepsilon_{cw}\;$$

In essence, equation (4) allows us to determine the dynamic nature of the relationship between time at home and child maltreatment referrals.

## Results

### Descriptive Statistics

**Figure 1.**
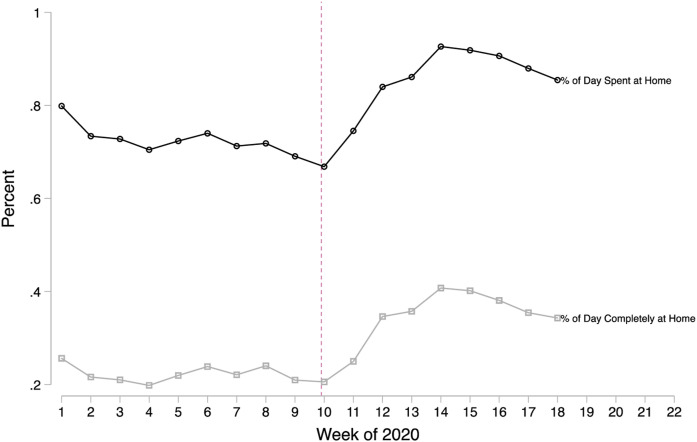
Trends in time at home in Georgia using cell phone tracking data. Source**:** Smartphone tracking data from SafeGraph, Inc weeks 1–18 of 2020 for the state of Georgia. Note**:** Georgia’s Governor declared a public health emergency due to COVID-19 for the state in the 10th week of 2020.

We begin by showing the immediacy with which Georgians responded to the Governor’s public health emergency declaration in 2020. Using cell phone tracking data from SafeGraph, Figure 1 shows large jumps in the time people spent at home, where the vertical line represents the week of the emergency declaration. Specifically, between weeks 10 and 14 of 2020, both the percent of the day that devices stayed home and the percent of the day devices stayed *completely at home* increased by 25.8 percentage points (39%) and 20.2 percentage points (98%) from pre-COVID levels. This equates to an average of 6.2 hours more per day at home. Following week 14, Georgians began reducing their sheltering-in-place prior to the state’s reopening plan which began on April 24, 2020 (week 16).

Within days of the public health emergency declaration, schools across Georgia were forced to close and transition to remote learning. Schools closed abruptly. The percent of students enrolled in K-12 schools who were affected by school closures jumped from approximately 4% of students on March 17 to 100% of students on March 18.

**Figure 2.**
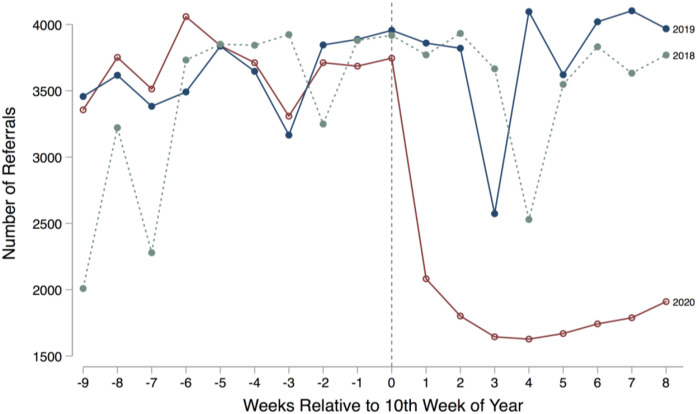
Raw trends in child maltreatment referrals in Georgia by week 2018–2020. Source**:** Administrative child maltreatment referral data for weeks 1–18, 2018–2020 from Georgia Division of Family and Children Services. Note**:** Georgia’s Governor declared a public health emergency due to COVID-19 for the state in the 10th week of 2020.

**Figure 3.**
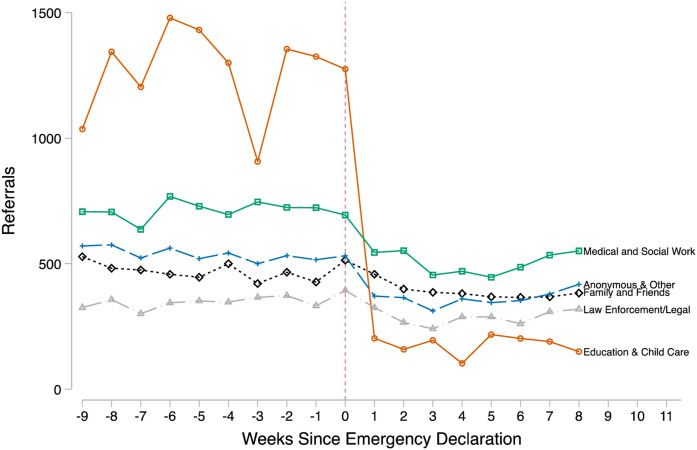
Raw trends in child maltreatment referrals in Georgia by reporter type. Source**:** Administrative child maltreatment referral data for weeks 1–18 from Georgia Division of Family and Children Services. Note**:** Georgia’s Governor declared a public health emergency due to COVID-19 for the state in the 10th week of 2020.

Next, Figure 2 displays raw trends in total referrals across the state of Georgia during the first 18 weeks of 2018, 2019, and 2020. In the week after the state’s emergency declaration and school closures, the number of referrals plummeted relative to 2018 and 2019 trends and remained the lowest of these 3 years for the duration of our study period. Around week 14, referrals begin to slowly increase, but never rebound to prior years’ levels. As shown in Figure 3, most of the 2020 drop is due to fewer referrals from education and childcare personnel. Other reporters, including medical professionals, social workers, family, friends, law enforcement, and anonymous reporters also reduced their referrals, but compared to education and childcare professionals, the magnitude was much smaller.^6^

### Effects of Georgia’s Emergency Declaration on Referrals

**Figure 4.**
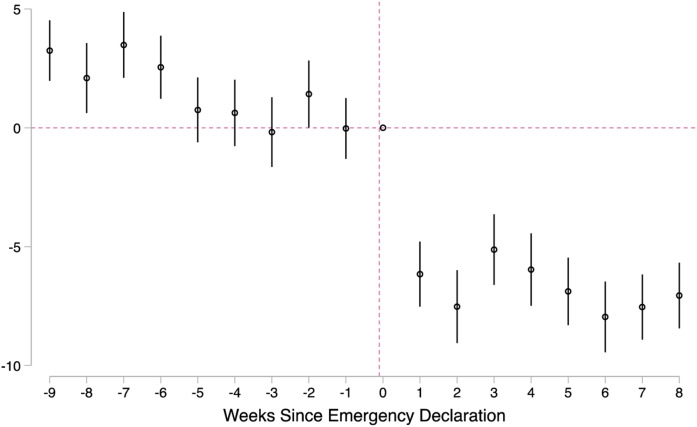
Effects of COVID-19 emergency declaration on child maltreatment referral rate. Source**:** Administrative child maltreatment referral data for weeks 1–18, 2018–2020 from Georgia Division of Family and Children Services. Note**:** Georgia’s Governor declared a public health emergency due to COVID-19 for the state in the 10th week of 2020. The outcome is the overall referral rate per 10,000 children. Model includes county, week, and year fixed effects. Robust SEs are clustered at the county-week level. 95% confidence interval is shown.

Descriptively, Figures 2 and 3 show clear and immediate drops in all types of referrals during the COVID-19 pandemic. Figure 4 offers a more robust measure of these changes, adjusting for county, week, and year fixed effects (as shown in equation (2)). This visual demonstrates several noteworthy points. First, in the 5 weeks leading up to the emergency declaration, there were no statistical differences between 2020 trends and trends in the previous 2 years. Following the emergency declaration, 2020 referrals decline precipitously, echoing Figures 1 and 2. The drop is immediate, and, during our study period, the adjusted models show that referral rates never fully rebound.

Table 1 provides the main estimates from equation (1) (Figure 4). This table also splits referrals by type of maltreatment alleged. Column 1 shows that after the emergency declaration in 2020 and relative to 2018 and 2019 trends, there were 8.17 fewer referrals per 10,000 children. Relative to the baseline mean rate of 14.01, this reduction represents a decrease of about 58%.

**Table 1.** Effects of COVID-19 Emergency Declaration on Maltreatment Referrals, by Maltreatment Type.

	Total referrals	Material neglect	Supervisory neglect	Educational neglect	Emotional neglect	Medical neglect	Physical abuse	Sexual abuse
Effect of emergency declaration	−8.17*** (0.26)	−0.60*** (0.05)	−3.51*** (0.16)	−0.56*** (0.03)	−0.50*** (0.03)	−0.25*** (0.02)	−1.61*** (0.06)	−0.43*** (0.03)
Mean Y in previous year	14.01	1.36	8.31	0.59	0.32	0.28	1.31	0.52
Relative % change	−58.3%	−44.1%	−42.2%	−94.9%	−156.3%	−89.3%	−122.9%	−82.7%
N	8586	8586	8586	8586	8586	8586	8586	8586

Source: Division of family and children services data weeks 1–18, 2018–2020. Notes: The outcome is the referral rate per 10,000 children. Regressions include county FE, week FE, and year FE. Robust standard errors are clustered at the county-week level. *p < 0.10, **p < 0.05, ***p < 0.01.

There is some variation in the magnitude of the reductions. After the emergency declaration there are 0.60 fewer material neglect referrals per 10,000 children, representing a 44% reduction. Declines in allegations of supervisory neglect are about the same size: 3.51 referrals per 10,000 children (42%). Allegations of emotional neglect, however, fall by approximately 156%. Educational and medical neglect referrals both decrease by approximately 90%. Meanwhile, physical abuse and sexual abuse referrals decline by 123% and 83%, respectively. Together, these results indicate that Georgia’s emergency declaration led to substantial and immediate decreases in all referral rates, with some differences by maltreatment type.

### Relationship Between Time at Home and Child Maltreatment Referrals

To describe the relationship between time spent at home during the pandemic and child maltreatment referrals, our next analysis combines the cell phone tracking data with the referral data (equation (3)). Table 2 shows that in the 8 weeks following Georgia’s emergency declaration, and using week 10 as the new baseline, a one percentage point increase in the percent of the day spent at home (approximately 15 minutes) is associated with 0.181 more referrals per 10,000 children. Relative to the baseline average in week 10 of 2020, this estimate represents a 1.2% increase.^7^ Overall, increasing referrals alleging material and supervisory neglect are driving this relationship, which increase by 3.5% and 1.0%, respectively. We do not detect a relationship between time at home and referrals for physical or sexual abuse or emotional, educational, or medical neglect across all counties.

**Table 2.** Relationship Between Time at Home and Maltreatment Referrals, by Maltreatment Type, 1–18th Weeks of 2020.

	Total referrals	Material neglect	Supervisory neglect	Educational neglect	Emotional neglect	Medical neglect	Physical abuse	Sexual abuse
All counties (*n* = 159)								
Percent of day spent at home*post	0.181** (0.082)	0.044** (0.017)	0.071* (0.040)	0.008 (0.008)	0.004 (0.009)	0.011 (0.008)	0.002 (0.020)	0.011 (0.012)
Mean Y in week 10 of 2020	14.950	1.269	7.387	0.619	0.591	0.443	2.067	0.918
Relative % change	1.2%	3.5%	1.0%	1.3%	0.7%	2.5%	0.1%	1.2%
N	2862	2862	2862	2862	2862	2862	2862	2862

Source: Division of family and children services data and SafeGraph data weeks 1−18 of 2020. Notes: The outcome is the referral rate per 10,000 children. Post = 1 for weeks 11–18 in 2020. Regressions include county FE and week FE. Robust standard errors are clustered at the county-level. *p < 0.10, **p < 0.05, ***p < 0.01

**Figure 5.**
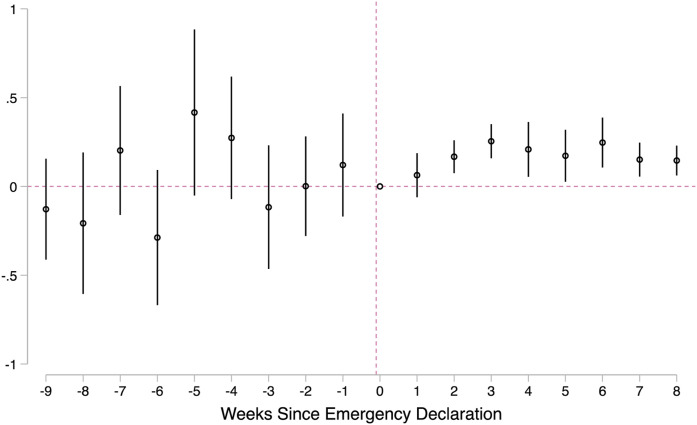
Dynamic relationship between time at home and total referrals. Source**:** Administrative child maltreatment referral data from Georgia Division of Family and Children Services and smart phone tracking data from SafeGraph, Inc for weeks 1–18 of 2020. Note**:** Georgia’s Governor declared a public health emergency due to COVID-19 for the state in the 10th week of 2020. The outcome is the overall referral rate per 10,000 children. Model includes county and week fixed effects. Robust SEs are clustered at the county-level. 95% confidence interval is shown.

Figure 5 reports results from equation (4). There appear to be dynamic effects in the role of time at home on referrals: notably, most of the effect transpires in the first 3 weeks following the emergency declaration. By the fourth week following the emergency declaration, the effect of time at home on referrals plateaus, though it is still positive and significant. Also important to note from Figure 5 is that prior to the emergency declaration the relationship between time at home and referral rates was nonsignificant; time at home during the COVID-19 pandemic has a *unique* relationship with child maltreatment referrals.

We also assess if the relationship between time at home and referrals varies across communities. In particular, we present estimates for counties by metropolitan and non-metropolitan status according to the United States Department of Agriculture (USDA) Rural-Urban Continuum Codes (i.e., areas with populations of 250,000 or more classified as metro), percent black residents, and poverty rates. We disaggregate the latter two characteristics by the median rate across all counties; that is, we consider a county whose poverty rates are above the median to have high poverty. In a county analysis, the rise in material neglect appears to be driven by non-metropolitan counties, counties with relatively fewer black residents, and higher-income counties. In contrast, the increase in supervisory neglect is driven by metropolitan counties and counties with relatively more black residents. Results are available upon request.

## Discussion

COVID-19 has increased risk factors associated with child maltreatment perpetration, such as unemployment, reduced income, alcohol abuse, intimate partner violence, and limited social support (Catalá-Miñana et al., 2017; Lindo, Schaller, & Hansen, 2018; Lowell & Renk, 2017; Schenck-Fontaine, Gassman-Pines, Gibson-Davis, & Ananat, 2017). Research from Florida (Baron et al., 2020), Indiana (Bullinger et al., 2020b), and Chicago, Illinois (Bullinger et al., 2021), suggests that child maltreatment allegations, substantiated cases of child maltreatment, and calls to 911 reporting child abuse, respectively, were lower than expected. In contrast, other data sources such as social media accounts and poison control records suggest that children were exposed to more violence and less supervision (Babvey et al., 2020; Chang et al., 2020). The Rape, Abuse, and Incest National Network reported an increase in calls from minors since March 2020 (Rape & Incest National Network RAINN).

The current research expands this burgeoning area of COVID-19 and child maltreatment research by using more fine-grained data on child maltreatment referrals, documenting trends by reporter type, studying how time spent at home, as measured by smartphone data, is associated with referrals, and examining differences across maltreatment types. Relative to 2018 and 2019, child welfare referral rates plummeted by approximately 58% following the emergency declaration. The largest drop in referrals was from mandated reporters working in education or childcare; this drop is expected given that these reporters had less access to observe children during Georgia’s lockdown. Notably, allegations of all types of child maltreatment declined, though emotional neglect and physical abuse referrals had the largest drops.

Given the substantial declines in referrals, an important finding of this study is the estimated relationship between time spent at home during the pandemic and maltreatment referrals. As time at home during the pandemic increased, the risk of material and supervisory neglect increased. The largest and most precise increases in material neglect occurred in non-metropolitan counties and counties with relatively fewer black residents. In contrast, the largest and most precise increases in supervisory neglect occurred in metropolitan counties and counties with relatively more black residents. These relationships are *in spite of* lower referral rates, overall.

Given the challenges in obtaining unemployment insurance (Goger et al., 2020) and the federal government’s delay in providing financial relief, the inability of parents to provide basic material goods for their children is unsurprising. Protection from economic stressors, such as eviction stoppages may have temporarily mitigated economic stressors (Han et al., 2020), but appear to have been insufficient or too late to eliminate the effect of economic hardship as a proximal cause of maltreatment. The relatively larger increases in material neglect in non-metropolitan counties and lower black population counties may additionally signal differences in pre-COVID social safety nets and access to services and other resources across urban and rural areas.

It is easy to speculate how increases in stress and time at home may impact supervision, especially for those managing working from home, providing care for multiple children who were previously in school or childcare, or where the primary caretaker had to go to work and leave their young children with someone unused to childcare or in less than desirable circumstances (Griffith, 2020). What is less understood is why families living in metropolitan counties and counties with more black residents might be at increased risk for supervisory neglect as compared to other counties. Other studies have found that domestic violence (including child abuse) was more likely to be reported in neighborhoods with more renters than homeowners, which may indicate the different experiences for those in especially tight quarters (Bullinger et al., 2021). For counties with more black residents, parents/caretakers may have been more likely to be frontline workers (Blau et al., 2021) leading to greater difficulty in finding appropriate, affordable, and available childcare when schools and daycares closed.

Supervisory neglect is difficult for CPS to address (Bullinger et al., 2020a; Fong, 2020), especially in the context of COVID-19 where there are limited resources to fix the novel issues facing families. This paper provides an early summary of the association between staying at home and child maltreatment referrals. This information will allow policy and decision makers to formulate policies and strategies to more effectively respond to their immediate, long-term, and post-pandemic needs.

### Policy and Program Implications

Findings from this work point to the need for a robust and differentiated social policy response to reduce the economic, social, and mental health stressors that are the sources of maltreatment.

Our work indicates that supervisory neglect increased as time at home also increased. This finding points to the need for greater resources to support parents and children *inside* their homes. For example, for parents who have transitioned to working from home, expanded access to paid family leave may help to reduce instances of supervisory neglect. For all working parents, expanded access to safe and affordable childcare is important, particularly for essential workers, frontline employees, and parents whose work cannot be performed remotely. Importantly, however, our findings indicate that *staying home* is problematic for supervisory neglect. This suggests that creative solutions to childcare and supervision for parents who are working from home during a pandemic are a worthwhile endeavor.

The study’s findings—especially those regarding time at home and increased neglect—have important implications for implementing clinical services and programs. More isolated areas and those that traditionally have a lower need for services may have fewer resources for families and additional attention may be needed in those areas. For example, evidence-based home visiting programs that directly target risk factors associated with parental neglect exist (Whitaker et al., 2020) and efforts are underway to expand the reach of these programs through a swift transition to virtual delivery (O’Neill, Korfmacher, Zagaja, & Duggan, 2020; Self-Brown, et al., 2020). Thus, broader implementation of clinical programming could offer increased availability and additional support to parents experiencing increased life and parental stressors, and could have substantial benefit.

### Study Limitations

This study has some limitations. First, our measure of adherence to staying at home is drawn from cell phone movement data and is essentially a measure of the intensity with which individuals stayed at home. As the smartphone data are aggregated and anonymous, we are also unable to link smartphone users to families much less to families those with risk of involvement with the child welfare system. It is a proxy for parent physical movement. Therefore, this research may fall victim to ecological fallacy and reduce our ability to infer causality. However, given that schools were closed, parents likely had higher than average compliance, which reduces this validity threat. Finally, although we know the number of hours that are spent at home, we cannot distinguish among the reasons why. Some people may have increased time at home because of job loss, while for others shifts to remote work increased time spent at home. This is significant because these distinct mechanisms likely require different policy responses.

Second, we use early release maltreatment data that are reported weekly at the county level. The data do not include detailed information on victims, reporters, or perpetrators and are referrals of child maltreatment. Referrals are a useful and valid indicator of child maltreatment risk (Drake, 1996); however, like all maltreatment data referrals, they have limitations such as the lack of substantiation. We note, however, that using aggregate data instead of individual-level data is not uncommon in child maltreatment research, and county-week level data are far superior to the more commonly used county-year or county-month, especially in the context of the COVID-19 pandemic, when changes were rapidly occurring within days and weeks.

Last, there is noise in child maltreatment reporting because not only must an incident occur but it also must be either observed or create some evidence (e.g., bruising) that can be observed by another person, *and* that observer must report the incident. We are unable to determine how much of the declines in referrals are from fewer opportunities for observation and detection or from reductions in true maltreatment. However, given that so many risk factors for maltreatment have been present during the pandemic, the likelihood that the declines are largely due to detection remains high. Despite these limitations, our study draws on innovative data to provide important insights about how children in Georgia, and likely elsewhere across the country, have fared during the pandemic.

## Conclusion and Future Directions

The global COVID-19 pandemic has markedly altered the lives of children and families. Our study sought to examine one important aspect of family life: the effect of COVID-19 policies intended to curb the spread of the virus on child maltreatment. We found substantial decreases in traditional sources of child maltreatment reports as a result of COVID-19 policies. We also found important increases in material and supervisory neglect in Georgia linked to increased time spent at home, along with other increases that vary by county metropolitan status and county demographics. That we did not find overall increases in other forms of child maltreatment is notable, particularly given the speculation regarding increases in physical abuse. It may be, however, that cases of physical and sexual abuse were missed since the child victims were not observed by a teacher in the same way and injuries were healed by the time the child interacted with someone outside their family. Future research should investigate this question taking into account the reductions in reporting.

In addition, it is unclear to what extent the social policy response to the pandemic, in the form of stimulus checks, increased unemployment benefits, and related policies, actively reduced child maltreatment. Future research should investigate the extent to which this package of economic stimulus policies affects child maltreatment.

Finally, as noted earlier, the data in this study offer several advantages over the current literature. They represent, however, the short-term effects of COVID-19 policies on child well-being. Understanding the implications and potential compounding effects of these experiences for children in the longer run, and post-pandemic environment, is of utmost importance.

## Notes

1 We refer to child maltreatment as an umbrella term including sexual abuse, physical abuse, and neglect (material, supervisory, educational, emotional, and medical).

2 As of this paper’s writing, the economy has recovered somewhat, but economists predict a slow recovery. Moreover, COVID-19 is ongoing. Future economic shutdowns may occur, making any recovery non-linear.

3 Common data sources for studying child maltreatment (e.g., formal child maltreatment reports to Child Protective Services (CPS) nationwide, medical records, and parent- and child-reported measures) are not yet available or are slowly becoming available to the research community. For example, the National Child Abuse and Neglect Data System (NCANDS): Child File usually releases data to the research community with a roughly 2-year lag.

4 For confidentiality reasons, SafeGraph excludes Census block groups with fewer than 5 devices. For a balanced panel, we drop block groups that are not in the panel every day during our time period.

5 For all counties, the ratio of devices to population is (on average) 53%. For 2/3rd counties, we have >50% of the population.

6 We do not have access to reporter type. The state agency provided us with the aggregate, state-level data by week.

7 On average, there are 31,470 phones tracked in each county-week. Therefore, one additional phone staying home all day would imply an increase of 0.00595 referrals per 10,000 children. This implies that if 168 additional cell phones stayed home all day, the referral rate would increase by 1 per 10,000 children.

## Appendix A

**Table:**

Maltreatment type	Description of allegation
Material neglect	Malnourishment/failure-to-thrive and inadequate food, clothing, and shelter
Supervisory neglect	Inadequate supervision; gunshot due to neglect; and suffocation/drowning due to neglect
Medical neglect	Inadequate health & medical care (e.g., delaying medical attention)
Emotional neglect	Emotional/psychological neglect
Educational neglect	Educational/cognitive neglect
Physical abuse	Fractures, dislocations, and sprains; suffocation/drowning due to abuse; Munchausen; gunshot due to abuse; intracranial or skull injury; spinal cord, nerve damage, subdural hematoma, internal chest, abdomen, and pelvic injury; lacerations, cuts, punctures, bruises, welts, abrasions, burns, and scalding; and poisoning
Sexual abuse	Exhibitionism/voyeurism; fondling; sodomy; penetration; genital injury; contraction of venereal disease; and sexual servitude/sex trafficking
Total allegations	All of the above plus family violence exposure; methamphetamine exposure; driving under the influence with a child under the age of 14 years; prenatal abuse/prenatal exposure/fetal alcohol spectrum disorder; and abandonment/rejection
